# Exploratory Analysis of the Impact of a Single Dose of Trastuzumab on the Immune Microenvironment in HER2-Positive Early-Stage Breast Cancer

**DOI:** 10.3390/biomedicines13112784

**Published:** 2025-11-14

**Authors:** Nikita Bastin, Jessica Mezzanotte-Sharpe, Rebecca Alvarez, Savannah C. Partridge, Suzanne M. Dintzis, Sasha E. Stanton, VK Gadi, Laura C. Kennedy

**Affiliations:** 1 School of Medicine, Vanderbilt University, Nashville, TN 37232, USA; 2 Department of Medicine, Medical Center, Vanderbilt University, Nashville, TN 37232, USA; 3 Department of Pathology, University of Washington, Seattle, WA 98195, USA; 4 Fred Hutchinson Cancer Center, Seattle, WA 98109, USA; 5 Department of Radiology, University of Washington, Seattle, WA 98195, USA; 6 Cancer Immunoprevention Laboratory, Earle A. Chiles Research Institute, Providence Cancer Institute, Portland, OR 97213, USA; 7 Division of Hematology and Oncology, University of Illinois Chicago, Chicago, IL 60612, USA; 8 Translational Oncology Program, University of Illinois Cancer Center, Chicago, IL 60612, USA

**Keywords:** tumor-infiltrating lymphocytes, trastuzumab, breast cancer, immune biomarker, tumor microenvironment

## Abstract

**Background:** How the tumor microenvironment (TME) influences treatment response in HER2+ breast cancer following HER2-directed therapy is crucial for individualizing therapies and is currently understudied. The purpose of this exploratory analysis was to elucidate changes in the TME following treatment with trastuzumab. **Methods:** Fourteen HER2+ early-stage breast cancer patients underwent tissue biopsies before and after a dose of trastuzumab. Samples were evaluated for stromal tumor-infiltrating lymphocytes (TILs) and RNA-based cell and gene expression signatures. Tumor inflammation signature scores were generated to measure whether an adaptive immune response developed to trastuzumab within the tumor. Patients were also stratified as immune responders or non-responders based on changes in TILs. **Results:** Of the 14 enrolled patients, 13 had samples available for analysis, and 7 had an immune response as assessed by changes in TILs compared to 6 non-responders. Trastuzumab treatment decreased PD-L1 and TGF-Beta signatures and increased CTLA4 gene signatures, although results were not statistically significant, and increased *DUSP1* expression. In the TIL responder group, there was increased expression of dendritic cells as well as *MARCO* expression. **Conclusions:** These findings, although exploratory in nature, highlight trastuzumab’s ability to induce an immune response and suggest that some patients may be more primed to mount an immune response following treatment than others. Patients without a robust response in TILs may benefit from additional agents to favorably modulate the TME for optimized responses to HER2-directed therapy, an area of research which warrants further study.

## 1. Introduction

Approximately 20% of all invasive breast cancers are driven by the human epidermal growth factor receptor 2 (HER2) oncogene as detected by gene amplification or protein over-expression [1]. This marker, previously associated with a worse overall prognosis and survival in patients with metastatic disease, is now an important therapeutic target for treating patients with both early-stage and metastatic disease. Trastuzumab, the first agent approved for targeting HER2, is a recombinant monoclonal antibody targeting HER2 that was shown to improve overall survival (OS) and progression-free survival (PFS) when combined with chemotherapy for the treatment of metastatic breast cancer [2]. Numerous studies also demonstrate trastuzumab’s efficacy in the early-stage setting, and the current standard-of-care treatment for patients with early-stage HER2-positive breast cancer is a combination of trastuzumab and cytotoxic chemotherapy with or without a second monoclonal anti-HER2 antibody, pertuzumab [3,4,5,6].

Currently, there is significant interest in tailoring therapy for patients with HER2-positive breast cancer by decreasing cytotoxic chemotherapy, as about a third of patients may achieve an excellent outcome with HER2-directed treatment alone without the addition of chemotherapy [7]. Several studies have shown that tumor-infiltrating lymphocytes (TILs) or immune-related signatures can be helpful in predicting trastuzumab sensitivity in patients [8]. In fact, Perez-Garcia et al. showed that patients with immune response-enriched tumors had greater pathological complete response rates when treated with neoadjuvant trastuzumab compared to those with non-immune response-enriched tumors. Despite the clinical use of trastuzumab for over 25 years, the immune mechanism of action is not clearly defined, though preclinical studies suggest both antibody-dependent cellular cytotoxicity (ADCC) and antibody-dependent cellular phagocytosis (ADCP) are involved [9,10,11]. Prior studies with available clinical samples that have assessed the tumor-immune landscape following treatment with trastuzumab and/or chemotherapy are limited [12].

Here, we present the results of RNA sequencing studies from an exploratory analysis in which HER2-positive early-stage breast cancer patients received tissue biopsies prior to and after receiving a single dose of neoadjuvant HER2-directed therapy with trastuzumab. The original goal of this study was to examine breast MRI as a tool to predict response and immune infiltration in response to trastuzumab treatment; correlative studies, reported here, explore trastuzumab-induced changes in the tumor microenvironment. Pre- and post-treatment tissue was collected for immune microenvironment assessment with TILs and RNA-based cell and gene expression signatures. In this study, immune responders were defined as having an increase in TILs of greater than one decile between the pre- and post-treatment timepoints, while non-immune responders had an increase of one decile or less between pre- and post-treatment timepoints. Although the sample size for this study was limited and the analysis is highly exploratory in nature, we report that trastuzumab appears to leverage pre-existing tumor microenvironment conditions to generate an immune response in tumors predisposed to developing an immune response, while also driving immune checkpoint expression and a decrease in anti-tumor cytokines in tumors that do not generate an immune response.

## 2. Materials and Methods

### 2.1. Patient Criteria and Enrollment

Patient criteria and enrollment for this study have been described previously [13]. Briefly, patients with a new diagnosis of early-stage HER2+ breast cancer planning for curative-intent treatment with breast tumor sizes greater than one centimeter were eligible for enrollment. Archival diagnostic biopsy tissue was used for the initial tissue sample, and post-treatment tissue was obtained through either a biopsy or definitive breast surgery two to four weeks after a single dose of run-in trastuzumab. Patients were permitted to receive pertuzumab in addition to trastuzumab as part of the run-in dose per the discretion of their treating provider.

### 2.2. Tumor Microenvironment Evaluation

Procedures involved in tumor microenvironment evaluation have been previously described but are reiterated briefly here [13].

### 2.3. TILs Assessment

Two trained clinical breast pathologists independently conducted separate assessments of stromal TILs on hematoxylin and eosin-stained slides from pre- and post-treatment tissue specimens. TILs were categorized into deciles for analysis, and the average of the two decile assessments was used to determine the final decile for analysis. The change in TILs was calculated by subtracting the post-trastuzumab TILs from the pre-trastuzumab TILs.

### 2.4. RNA Isolation

Total RNA was extracted from unstained slides using the Qiagen AllPrep DNA/RNA formalin-fixed paraffin-embedded kit (Qiagen, Germantown, MD, USA). Slides were deparaffinized, washed with 100% ethanol and nuclease-free water, and air-dried. Sections were then coated with 3% glycerol and scraped into a polymerase chain reaction tube, after which RNA extraction was performed.

### 2.5. Immune Signature Scores

Immune signature scores were obtained through a commercially available service (Nanostring, Seattle, WA, USA) using the Nanostring PanCancer IO360 panel. Samples were submitted to Nanostring for gene expression profiling, and samples that failed quality control metrics were excluded from analysis.

### 2.6. Statistical Analysis

The primary objective of this study was to assess trends in immune responses two weeks after a single dose of HER2-directed therapy with trastuzumab (or trastuzumab and pertuzumab). Pre- and post-treatment stromal TIL levels for the cohort were compared using a paired *t*-test. Genes were normalized using a ratio of the expression value to the geometric mean of the housekeeping genes on the panel. For tumor inflammation signature (TIS) genes, genes in the TIS are normalized using a ratio of the expression value to the geometric mean of the housekeeper genes used only for the TIS. The housekeeper-normalized data was Log (2) transformed. Immune signatures were adjusted with constants to express data in a similar range. Differential expression was fit on a per-gene or per-signature basis using a linear model for analyses without a blocking factor. The statistical model uses the expression value or signature score as the dependent variable and fits a grouping variable as a fixed effect to test for differences in the levels of that grouping variable.

Expression (gene or signature) = µ + Group + ε

*p*-values are adjusted within each analysis, gene, or signature, and on the grouping variable level difference *t*-test using the Benjamini and Yekutieli False Discovery Rate adjustment to account for correlations amongst the tests. All models were fit using the limma package in R [14,15,16].

Mean immune signatures among low TIL responders and high TIL responders were calculated and compared using paired two-tailed heteroscedastic *t*-tests. This analysis was performed using samples obtained both prior to and following trastuzumab treatment. Mean gene signature scores amongst samples were compared using the same technique.

## 3. Results

### 3.1. Patient Demographics

Patient demographics are included in Table 1. Fourteen women (median age 52 years, range 37–69 years) diagnosed with invasive ductal carcinoma were enrolled in the study between January 2013 and September 2019. Clinical characteristics of the cohort have been previously described [13]. Briefly, three of the patients had biopsy-proven lymph node involvement at time of diagnosis; these patients proceeded with a full course of neoadjuvant chemo- and HER2-directed therapy after the study window was complete (following the run-in dose of trastuzumab). Half of the patients were estrogen receptor (ER)-positive (i.e., Allred score 3/8 or greater). For this exploratory analysis, as previously described in [13], an immune response was defined by changes in TILs: an increase greater than one decile was considered an “immune responder,” and “immune non-responders” had either no change in TILs or a change of one decile or less. Based on this definition, there were seven responders and six non-responders; patient 8 was excluded from analysis due to a follow up sample that was unable to be quantified for TILs. Patient 8 also did not have data generated from immune signature scores and so was excluded from the rest of this study’s analysis.

### 3.2. Longitudinal Cell Signatures and Gene Expression

**Figure 1:**
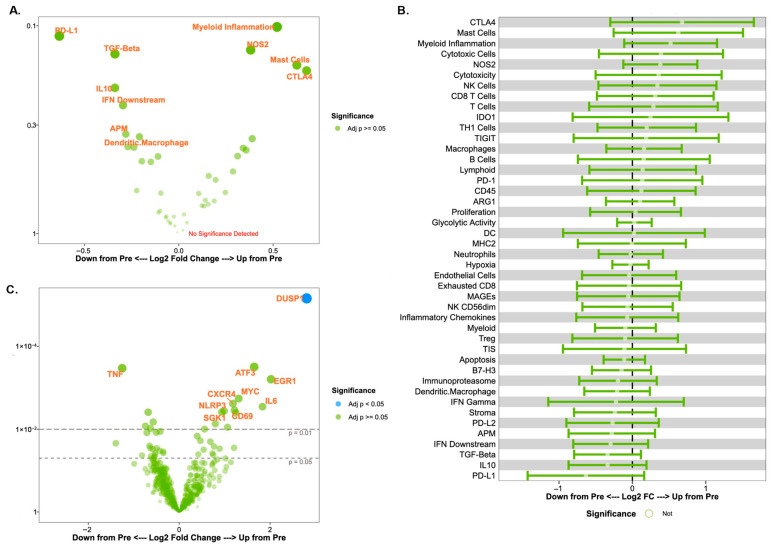
Changes in immune signatures and gene expression over time across all patients. (**A**) Volcano plot of cell signature scores after a single dose of trastuzumab (post-treatment vs. pre-treatment). For (**A**,**C**), each signature (**A**) or gene expression (**C**) difference between timepoints is represented along the x-axis, with the *p*-value along the y-axis. Signatures or gene expressions with greater statistical significance appear higher on the plot and are larger and darker in hue. Signatures or genes with greater differential expression from baseline are further from the plot center, with rightward values representing an increase in expression and leftward values representing a decrease in expression relative to baseline. Of note, the y-axis values (*p*-values) for (**A**,**C**) are highly exploratory in nature. (**B**) Forest plot of cell signature scores after a single dose of trastuzumab (post-treatment vs. pre-treatment). (**C**) Volcano plot of gene expression patterns after a single dose of trastuzumab.

To investigate the change in immune microenvironment following treatment, tissue biopsy samples were obtained before and after the administration of trastuzumab as previously described [13]. We initially focused on changes in cell signatures and gene expression scores over time in the entire cohort. A volcano plot and forest plot of the changes in cell signatures over time are shown in Figure 1A and Figure 1B, respectively. A volcano plot of the changes in gene expression scores over time is shown in Figure 1C. Of note, these findings are provided for descriptive and exploratory purposes only. The study’s small sample size has a risk of sampling variability affecting results rather than a true biologic effect, so results must focus on the qualitative patterns rather than statistical significance. We observed trends towards decreased PD-L1 (*p* = 0.11) and TGF-Beta (*p* = 0.14) signatures, and increased CTLA4 signatures (*p* = 0.16) after treatment with trastuzumab. There was a significant increase in dual specificity phosphatase 1 (*DUSP1*) expression after trastuzumab treatment (*p* < 0.001) in both the immune responder and non-responder groups. The confidence interval reported in log change was (1.81, 3.79).

### 3.3. Patient and Pathologic Features in Immune Responders and Non-Responders

**Figure 2:**
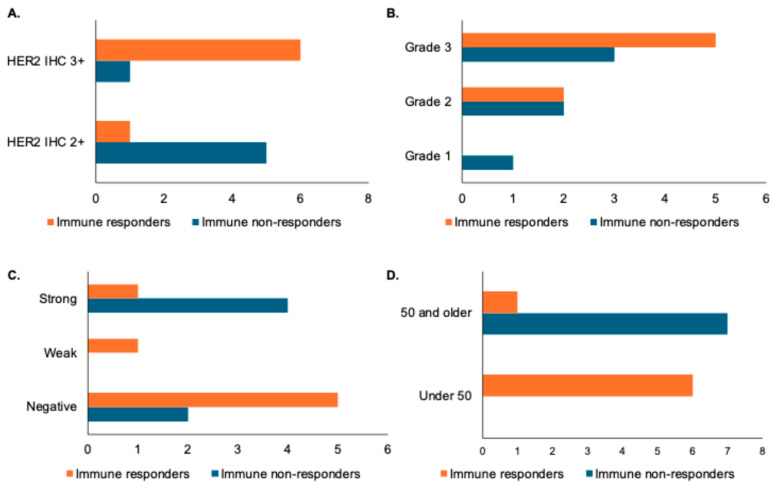
Association of pathologic and patient features with immune response as defined by changes in stromal TILs. X-axes for panels (**A**–**D**) represent number of patients. (**A**) Bar graph showing distribution of HER2 IHC in immune responders (increase in stromal TILs of greater than one decile) versus immune non-responders. (**B**) Bar graph showing histologic grade distribution in immune responders versus non-responders. (**C**) Bar graph showing distribution of estrogen receptor (ER) status by Allred score (with a weak distribution indicated by a score of 3–6 and a strong distribution indicated by a score of 7 or 8) in immune responders versus non-responders. (**D**) Bar graph showing age distribution in immune responders and non-responders.

We next examined patient and pathologic features among immune responders and non-responders as assessed by changes in TILs following trastuzumab treatment. Samples with HER2 IHC 2+ were noted to be more prevalent among low TIL responders, while HER2 IHC3+ was more prevalent among high TIL responders (Figure 2A). A patient with grade 1 disease was noted to be an immune non-responder, but there were no differences in grade 2 or grade 3 disease among immune responders and non-responders (Figure 2B). Low TIL responders appeared to be distributed evenly between ER-positive and ER-negative samples, while immune responders were predominantly ER-negative or had weak ER expression (Figure 2C). Finally, age appeared to correlate with immune response as assessed by change in TILs, with patients under 50 more likely to have a change in TILs than patients over 50 (Figure 2D). It is important to note, however, that none of these changes were statistically significant when assessed by a paired Student’s t-test, likely due to the small overall sample size, so larger studies are needed to determine if any of these features truly correlate with TIL response.

### 3.4. TIL Changes Post-Trastuzumab Administration

**Figure 3:**
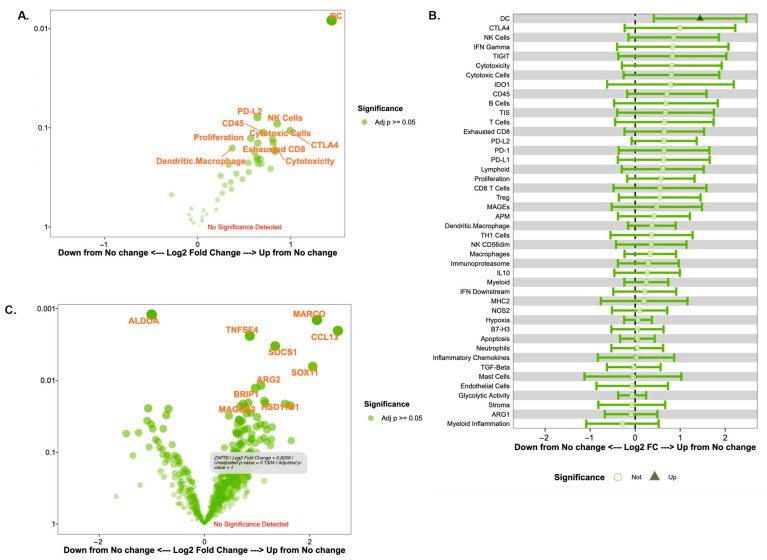
Immune signature and gene expression changes in immune responders versus non-responders as defined by changes in stromal TILs. (**A**) Volcano plot of immune cell signatures in TIL responders versus non-responders by fold change (difference on Log(2) scale on x-axis) and significance (*p*-value) on the y-axis. For (**A**,**C**), signatures or genes that have greater statistical significance appear higher on the plot with larger, darker points, while signatures or genes that have a greater differential expression appear further from the center of the plot. Genes further to the right indicate an increase in expression, while genes further to the left indicate a decrease in expression relative to the baseline group. Of note, the y-axis represents *p*-values that are for exploratory analysis only. (**B**) Forest plot of cell signatures in TILs responders versus non-responders. (**C**) Volcano plot of gene expression patterns based on a change in TIL responders versus non-responders.

A volcano plot and a forest plot of cell signatures comparing immune responders and non-responders are shown in Figure 3A and Figure 3B, respectively. A volcano plot of gene expression scores is shown in Figure 3C. Again, these results must be interpreted with caution and with a focus on general trends rather than statistical significance given the study’s small sample size. Although we report *p*-values, these must be interpreted with caution, as we detail in our study’s limitations. Differential expression analysis revealed that between clinical samples with immune responders compared to immune non-responders, there was significantly increased expression of dendritic cells in the immune responder group (*p* < 0.01). The confidence interval reported in log change was (0.41, 2.5). There was also an increased expression of several other cell signatures in the immune responder group with potential biologic effects: natural killer (NK) cells (*p* = 0.09), PD-L1 (*p* = 0.21), PD-L2 (*p* = 0.08), CTLA-4 (*p* = 0.11), and cytotoxic cells (*p* = 0.13). In terms of gene expression changes, there was increased gene expression of *MARCO* (*p* < 0.01), a scavenger receptor found on a subset of tumor-associated macrophages, and *CCL13* (*p* < 0.01), a chemokine that serves as a chemoattractant for monocytes and T cells, and decreased expression of *ALDOA* (*p* < 0.01) following trastuzumab treatment (Figure 3C).

## 4. Discussion

The goal of this study was to examine the utility of breast MRI as a non-invasive biomarker to monitor the immune response to HER2-directed therapy in patients with early-stage HER2-positive breast cancer [13]. In the original analysis, RNA sequencing generated immune signature scores that provided a comprehensive evaluation of the immune response in an individual tumor [13]. In this exploratory analysis, we further explore the results of that RNA sequencing data and report changes in stromal TILs in pre- and post-trastuzumab treatment. To our knowledge, this study is distinct from previous studies because patients were solely treated with HER2-targeted monoclonal antibodies and did not receive cytotoxic chemotherapy prior to analysis, which might impact immune response; many prior studies included combination chemotherapy and trastuzumab [9,10]. One clinical study assessed breast cancer response to trastuzumab alone and noted that the tumor microenvironment immune composition following trastuzumab treatment may mediate the response to therapy [12]. Our study also reports data from clinical samples, while most existing data assesses the tumor-immune microenvironment using preclinical samples [9]. In addition to reporting global changes in immune signature scores, we also divided patients into those who experienced TIL infiltration in response to trastuzumab treatment (immune responders) and patients who did not (immune non-responders). Multiple prior studies have demonstrated that high levels of stromal TILs pre-treatment or immune infiltration in response to treatment is a positive prognostic marker [8,17,18,19,20,21,22,23].

While bulk enumeration of TILs is a clinically validated marker and therefore can separate potential immune responders from non-responders, it provides limited insight into tumor microenvironment dynamics. TIL subsets also may be either pro-tumorigenic or anti-tumorigenic. These are the limitations of using changes in TILs as a marker of overall tumor microenvironment changes in this study. To further characterize the tumor-immune infiltrate, we determined RNA-based immune signatures pre- and post-trastuzumab. While the tumors with TIL responses did have an overall increase in the total number of TILs after a single trastuzumab dose, the composition of the TIL subsets did not change profoundly pre- vs. post-treatment. The main difference between immune responsive vs. non-responsive TMEs seemed to be higher immune signature scores for dendritic cells, NK cells, and cytotoxic cells in the pre-treatment sample, although only the dendritic cell signature was statistically different between the two groups. Trastuzumab has been shown to facilitate the uptake of HER2 by dendritic cells, thus enabling more efficient expansion of antigen-specific cytotoxic T cells [9]. Other studies have further noted that the clinical efficacy of trastuzumab correlates with the presence of NK cells [24]. In fact, patients whose immune effector cells, including NK and dendritic cells, can bind more tightly to the Fc domain have been observed to have stronger responses to trastuzumab [25].

For single gene expression analysis, there were two notable genes with potential biologic effects. We observed that immune responders had higher expressions of *MARCO*, a gene expressing a pattern recognition receptor on tumor-associated macrophages. While the mechanism of MARCO is unknown, its upregulation has been noted to be a likely immune evasion mechanism from tumor cells [26]. *MARCO* has further been revealed to be a gene overexpressed in the tumor microenvironment and linked to poor prognosis in breast cancer [27]. However, there have also been analyses from the TCGA database that suggest increased *MARCO* expression is associated in many cancer types with increased checkpoint molecule expression despite its association with poor prognosis, suggesting that it may be marker of immunotherapy-responsive tumors [28]. Additionally, we observed increased *DUSP1* expression after trastuzumab in all patients, suggesting that this is a change that is driven by the trastuzumab regardless of the TME. *DUSP1* encodes for dual-specificity protein phosphatase 1, which dephosphorylates, and therefore inactivates, multiple MAPK kinases, allowing DUSP1 to regulate MAPK activity during the cell cycle [29]. DUSP1 has been noted as a key downstream target of HER2 but notably was found to be increased in anti-HER2 therapy resistant cells, perhaps showing an early attempt at developing resistance to trastuzumab in our patient samples [30]. In vitro models show that combined inhibition of MKP1 and HER2 actually leads to enhanced cell death in breast cancer cells, which is consistent with other studies showing that combined approaches targeting both HER2 and DUSP1 were more efficacious than those targeting HER2 alone [30]. This is a novel and promising treatment combination that warrants further study.

Interestingly, we also noted that only patients under the age of 50 had an immune response as assessed by TIL infiltration. Although not statistically significant, larger studies are needed to determine if baseline characteristics will be able to help identify patients in the future who may be able to deescalate neoadjuvant chemotherapy in favor of using HER2-targeted antibody therapy alone, given their robust immune responses to treatment.

### 4.1. Limitations

This study has several limitations. The greatest limitation is the small number of patients, which limits the generalizability of the study findings as well as the robustness of the statistical inferences. Although we have a large number of analyzed variables in terms of the gene and immune signature scores, the small sample size regarding the actual number of patients in this study makes our statistical conclusions less reliable and prone to potential false positive results from multiple testing of the gene and immune signature scores. Additionally, while we have presented volcano and forest plots in Figure 1 and Figure 3, we must acknowledge that the *p*-values and confidence intervals presented in those plots are unstable due to our small sample size, and the significance represented by these results may potentially reflect sampling variability rather than true biological effects. As we state in the Section 3, these figures are presented for descriptive and exploratory purposes only. It is also important to note that the classification of immune responders and non-responders in this study only utilizes change in TILs as assessed by changes in deciles, which is not a perfect marker for the entire tumor-immune microenvironment but instead represents one immune compartment of the overall microenvironment. Presented results should therefore be interpreted with caution and should be used as preliminary data for future large-scale cohort studies rather than definitive conclusions from this work.

The recruitment period for this study also lasted six years from 2013 to 2019 and was slow accruing due to clinical workflows. Despite the long enrollment period, all procedures for patient selection and tissue processing/analysis were consistent over the course of this study. Of note, the enrolled patients did have variabilities in tumor grade, ER status, and disease grade, as noted in Figure 1. This represents a potential source of biological variability that may influence gene expression patterns and therefore contribute to differences within the groups, again potentially increasing variability and limiting the study results.

An additional limitation of this work is that we were only able to use a single biopsy site, which may fail to reflect tumor heterogeneity. In addition, the patients in this study did not undergo identical treatments, limiting our ability to correlate patient outcomes with our findings. Again, larger studies are needed to confirm the findings from this study.

### 4.2. Conclusions

Despite the limitations from the small sample size, this study provides intriguing preliminary data regarding the changes in the immune microenvironment of breast tumors following trastuzumab treatment. This expands upon the previous literature in this area and will further guide the development of larger-scale studies exploring personalized therapeutics in HER2-positive breast cancer.

## Figures and Tables

**Table 1:** Patient demographics and tumor pathologic features. IHC: immunohistochemistry. sTILs: stromal TILs. UNK: unknown. N/A: not available. Green rows represent immune responders; blue rows are immune non-responders.

Patient ID	Age at Diagnosis (Years)	ER Status (Allred Score)	Combined Histologic Grade	HER2 IHC	HER2 Copy Number	HER2:CEP17 Ratio	Ki67	sTILs Pre (Decile)	sTILs Post (Decile)
1	41	Negative	2	2+	11.6	6	65	1	3.5
2	39	Negative	3	3+		N/A	75	6	9
3	37	Negative	3	3+		N/A	50	0	9
4	57	Negative	2	3+		N/A	10	1.5	7
5	40	Negative	3	3+		N/A	UNK	1.5	3
6	60	Negative	1	2+	35	13	UNK	1	1
7	61	Negative	3	3+		N/A	50	1	1
8	53	Positive (3/8)	2	3+		N/A	40	5.5	UNK
9	47	Positive (4/8)	3	3+		N/A	UNK	3	8.5
10	37	Positive (7/8)	3	3+		N/A	38	3	7
11	64	Positive (8/8)	2	2+	7.95	3.1	UNK	1	1
12	69	Positive (8/8)	3	2+	6	1.8	UNK	1	1
13	51	Positive (8/8)	3	2+	4.33	2.83	30	1	2
14	52	Positive (8/8)	2	2+	4	3.3	UNK	1	1

## Data Availability

Pathologic and clinical data from this study may be made available upon request to the corresponding author.
